# Co‐treatment with erythropoietin derived HBSP and caspase‐3 siRNA: A promising approach to prevent fibrosis after acute kidney injury

**DOI:** 10.1111/jcmm.70082

**Published:** 2024-12-04

**Authors:** Yuanyuan Wu, Yanan Wang, Fei Chen, Cheng Han, Lili Huang, Wenli Sai, Yaping Fan, Nigel J. Brunskill, Bin Yang

**Affiliations:** ^1^ Department of Pathology Medical School, Nantong University Nantong China; ^2^ Department of Nephrology, Leicester‐Nantong Joint Institute of Kidney Science Affiliated Hospital of Nantong University, Medical School of Nantong University Nantong China; ^3^ Department of Critical Care Medicine Affiliated Hospital of Nantong University Nantong China; ^4^ Department of Cardiovascular Sciences University of Leicester, University Hospitals of Leicester NHS Trust Leicester UK

**Keywords:** acute kidney injury, caspase‐3, helix B surface peptide, renal fibrosis, siRNA

## Abstract

Acute kidney injury (AKI) is a risk factor of chronic kidney disease, without specific treatment. This study investigated the effect of co‐treatment using erythropoietin‐derived helix B surface peptide (HBSP) and caspase‐3 small interfering RNA (CASP3siRNA) on preventing fibrosis post AKI in order to achieve better efficacy by different action mechanisms. Ischemia–reperfusion (IR) in mice was induced by clamping bilateral renal pedicles for 30 min followed by 2‐week reperfusion, with HBSP and/or CASP3siRNA administered at the onset of IR. Serum creatinine, apoptosis, active caspase‐3 and high mobility group protein B1 (HMGB1) in kidneys were decreased by HBSP, CASP3siRNA or both, with increased PCNA. α‐SMA expression and collagen I deposition were also reduced by CASP3siRNA and both. Most interestingly, the co‐treatment further reduced tubulointerstitial damage and fibrosis, but raised PCNA compared to CASP3siRNA. EPOR/βcR was reduced by HBSP, and positively correlated with Sirius red staining, whereas EPOR was unchanged. In TCMK‐1 cells, H_2_O_2_ raised apoptosis and α‐SMA were reduced by HBSP, while the same was occurred to HMGB1. However, HMGB1 was further increased by EPOR siRNA under H_2_O_2_ stimulation with/without HBSP treatment. In conclusion, this study demonstrated synergistic long‐term renoprotection post IR‐AKI by HBSP and CASP3siRNA, which may be due to co‐inhibiting inflammation and stimulating repair at early stage, and subsequently preventing fibrosis.

## 1 INTRODUCTION

Chronic kidney disease (CKD) is characterized by the progressive and irreversible decline in kidney function, with critical pathological features of tubular atrophy and interstitial fibrosis that lead to end‐stage renal disease.^1^ Acute kidney injury (AKI) caused by ischemia–reperfusion (IR) injury contributes one of major etiologies of CKD development.^2^ Mechanistic studies have revealed that manipulating cell death pathways not only preserves kidney parenchymal cells and limits inflammation, but also improves the recovery of the IR injured kidneys.^3^, ^4^ However, therapeutic strategies that balance renal injury and repair, thus preventing the progression of AKI to CKD, are still under investigation.

Regulated cell death including apoptosis and necroptosis has emerged as a central event in the progression of AKI. Kidney tubular epithelial cells (TECs) are most vulnerable to IR injury, and undergo apoptosis that contributes to the chronic progression of AKI and fibrosis.^5^, ^6^ Targeting apoptotic cell death of TECs, a critical element in initiating progressive injury, has shown promise in preventing the transition from AKI to CKD.^7^, ^8^ The activation of caspase‐3 is the central step of apoptosis execution.^9^ Silencing caspase‐3 using small interfering RNA (CASP3siRNA) has been shown to protect auto‐transplanted kidneys by reducing systemic inflammation and interstitial collagen deposition in a 2‐week porcine model, indicating caspase‐3 as a critical target in preventing the progression of AKI to CKD.^10^

In response to renal IR injury, an innate repair mechanism is initiated by rapidly raising a heterodimer receptor composed of erythropoietin (EPO) receptor and *β* common receptor (EPOR/βcR) on surface of TECs.^11^ A large dose of EPO is necessary for the activation of EPOR/βcR, as it has higher affinity for the erythropoietic homodimer (EPOR)_2_ than the heterodimer receptor.^12^, ^13^ However, therapeutic dose of EPO for renoprotection could result in hypertension^14^ and thrombosis,^15^ as well as inducing renal fibrosis as a long‐term side effect.^16^ Helix B surface peptide (HBSP) derived from EPO selectively binds to EPOR/βcR and not the homodimer (EPOR)_2_, so it is renoprotective, but not erythropoietic.^17^, ^18^ The administration of HBSP at the onset of IR injury has been shown to prevent apoptotic cell death and the release of high mobility group protein B1 (HMGB1), as well as correct maladaptive repair characterized as excessive deposition of interstitial collagen through activating PI3K/Akt/FoxO3a pathways.^19^, ^20^, ^21^

In this study, we demonstrated that early co‐treatment with CASP3siRNA and HBSP further preserved kidney structure, promoted proliferation and reduced collagen deposition than single CASP3siRNA treatment at 2 weeks after bilateral kidney IR. Therefore, the co‐treatment of CASP3siRNA and HBSP represents a promising novel therapeutic approach to balance injury and repair in kidneys, and then mitigate the progression of AKI to CKD.

## 2 MATERIALS AND METHODS

### 2.1 Mouse renal IR model

Adult (8–12 weeks) WT male C57BL/6 mice were obtained from the Experimental Animal Center of Nantong University, China. Animal maintenances and experiments were conducted in accordance with the guidelines of the Laboratory Animal Monitoring Committee of Jiangsu Province. Mice were randomly divided into 7 groups: (1) Sham (*n* = 5); (2) IR (*n* = 9); (3) IR + CASP3siRNA (*n* = 8); (4) IR + NCsiRNA (*n* = 6); (5) IR + HBSP (*n* = 7); (6) IR + HBSP + CASP3siRNA (*n* = 7); (7) IR + HBSP + NCsiRNA (*n* = 8). IR animals were subjected to bilateral kidney ischemia for 30 min and reperfusion for 2 weeks. The number of mice in each group was determined using a power calculation based on the change of the key parameter in our previous IR models^19^ and CHBP intervention study.^22^ The animals were anaesthetised by intraperitoneal injection of pentobarbital sodium. Dorsal incisions were used to isolate both kidneys sequentially and then bilateral renal pedicles were occluded with non‐traumatic vascular clamps for 30 min. The kidneys were observed until patchy blanching developed and then replaced into the abdominal cavity. The clamps were subsequently removed and the surface colour of the kidney gradually returned to normal pink from dark red, confirming the effectiveness of ischemia and reperfusion process. The sham animals underwent a similar procedure, except for the clamping of renal pedicles.

### 2.2 Therapeutic intervention

HBSP, a linear peptide with the 11 amino acid sequence QEQLERALNSS, was synthesized by Shanghai Science Peptide Biological Technology, China. The molecular weight of HBSP was 1274.33 Dalton. HBSP was dissolved in saline and administered via intraperitoneal injection at two identical doses (24 nmol/kg in total). The first dose was given at the onset of ischemia, and the second dose was administered 15 min after reperfusion. CASP3siRNA targeting murine caspase‐3 mRNA and a negative control siRNA (NCsiRNA, Ambion in vivo) was synthesized with chemical modification of locked nucleic acid.^23^ The siRNAs were injected into the tail vein of the mice 2 h before the IR surgery at a dose of 0.03 mg/kg, dissolved in saline.

### 2.3 Cell culture

TCMK‐1 cells, a mouse kidney epithelial cell line (ATCC: CCL‐139), were maintained in the DMEM/F12 1:1 medium (Gibco, Paisley, UK) supplemented with 10% fetal bovine serum (Sigma, Dorset, UK), at 37°C in a 5% CO_2_ humidified incubator. The cells were seeded into six‐well plates at 1 × 10^5^ cells/well before treated with 100 μM H_2_O_2_ to mimic oxidative stress during kidney IR the next day. At the same time, some wells were also treated with HBSP at 5, 10, 20 or 40 ng/mL dissolved in PBS. For the cells treated with small interfering RNA targeting EPOR (EPORsiRNA, s65611, Thermo Fisher Scientific, Rockford, USA) or its negative control (NCsiRNA, 4390843, Thermo Fisher Scientific), 20 nM siRNAs were transfected into the cells 6 h before H_2_O_2_ and/or HBSP intervention under the help of Lipofectamine RNAiMAX (Invitrogen, Carlsbad, USA) according to the manufacturer's instruction. After treatment for 24 h, cells were collected to determine the percentage of early and later apoptotic cells by Annexin V/PI staining (Roche, Mannheim, Germany) on a flow cytometer (FACSCanto, BD, Bergen, USA). For some cells treated for 24 or 48 h, cell lysis was performed using RIPA buffer (Beyotime, Shanghai, China) for western blotting. At least three independent experiments were performed with two replicates each time.

### 2.4 Blood biochemistry

A QuantiChrom™ Creatinine Assay Kit (BioAssay Systems, Hayward, USA) was applied to determine the serum creatinine (SCr) level of each mouse. Thirty microlitre of each concentration of standard or serum sample (diluted by five folds with sample buffer) were applied and the procedure and calculations were performed according to the manufacturer's instructions. The detection was done three times independently.

### 2.5 Histological assessment

The paraffin‐embedded kidney tissues were processed to sectioning and staining by haematoxylin & eosin to assess cortical tubulointerstitial damage (TID). The stained sections were semi‐quantitatively scored for tubular damage (vacuolation and detachment from basement membrane), cell and cell debris in lumina and interstitial expansion (edema and infiltrations) in each field, based on a scale of 1–4. In each observed field, the damage affecting 0%–5% of the area was scored 1; damage affecting 5%–25% of the area was scored 2; damage affecting 25%–75% of the area was scored 3; and damage exceeding 75% of the area was scored 4. Two researchers blindly reviewed all sections independently. In each section, 12 randomly selected cortical fields were scored at 200× magnification. The average score from all observed fields in both left and right kidneys of each animal was used for the calculation of the final score of each group.

### 2.6 TUNEL staining

The TUNEL Apoptosis Detection Kit (Millipore, MA, USA) was used for staining the apoptotic cells in the kidney cortex according to the manufacture's instruction. Briefly, kidney paraffin sections were digested with 20 μg/mL proteinase K for 10 min at 37°C, incubated with terminal deoxynucleotidyl transferase, anti‐digoxigenin‐peroxidase sequentially and developed with 3‐amino‐9‐ethylcarbazole (AEC, dark red colour). Apoptotic cells were examined in the cortical tubulointerstitial areas at 400× magnifications in up to 20 fields by two researchers blindly and independently. The average number of positively stained cells from all observed fields in both left and right kidneys of each animal was used for the calculation of the final data of each group.

### 2.7 Western blotting

Total protein from the kidney tissue or TCMK‐1 cells was extracted and 25 μg of each protein sample was separated in reduced sodium dodecyl sulfate‐polyacrylamide gel and then wet transferred to polyvinylidene difluoride membranes. The membranes were then blocked with 5% (weight/volume) fat‐free milk, followed by incubation with an anti‐mouse full length caspase‐3 antibody (1:400 dilution, CST, Danvers, USA), or an anti‐mouse high mobility group box 1 (HMGB1) antibody (1:1000 dilution, CST), or an anti‐ mouse PCNA antibody (1:1000 dilution, DAKO, Glostrup, Denmark) or an anti‐mouse β‐actin antibody (1: 8000 dilution, Abcam, Cambridge, UK), or an anti‐mouse α‐SMA antibody (1:1000 dilution, Abcam), or an anti‐mouse EPOR antibody (1:1000 dilution, Abnova, Taiwan), an anti‐mouse GAPDH antibody (1:1000 dilution, CST) or an anti‐mouse Tubulin antibody (1:1000 dilution, CST) at 4°C overnight. The membranes were then probed with horseradish peroxidase‐conjugated goat‐anti‐mouse/rabbit antibodies (1:20 dilution, DAKO) and the blots were visualized using the enhanced chemiluminescence detection system (Pierce™ ECL, Thermo Fisher Scientific). Band intensities were quantified by Image Lab 2.0 software (Bio‐Rad). The relative protein abundance in each sample was first normalized to that of β‐actin, GAPDH or Tubulin, and then a ratio of each group to sham group was calculated. The experiment was performed three times independently.

### 2.8 Co‐immunoprecipitation

The detailed method of co‐immunoprecipitation was described previously.^24^ Briefly, EPOR protein in kidney tissue homogenates were recognized and complexed by anti‐mouse EPOR antibody (Abnova) during overnight incubation at 4°C on a rotator. Afterwards, the antigen–antibody complex was pulled down by protein A sepharose beads (GE healthcare, Pittsburgh, USA). The βcR protein that formed heterodimer receptor with EPOR could also be precipitated with the beads, which was revealed by immunoblotting with anti‐βcR antibody (Santa Cruz, Dallas, USA). Thus, the level of βcR protein detected represents the corresponding level of EPOR/βcR.

### 2.9 Immunohistochemistry staining

The kidney paraffin sections were de‐waxed and digested with proteinase K 40 μg/mL for 15 min before blocking with 10% goat serum diluted in PBS for 30 min. Thereafter, the sections were incubated with Rabbit‐anti‐mouse collagen I (1:100 dilution in 1% goat serum, Abcam) in a humidity chamber at 4°C overnight. For negative controls, normal rabbit immunoglobulin G was applied at the same concentration of primary antibody. The sections were then incubated with an HRP conjugated secondary antibody (DAKO, Glostrup, Denmark), before developing with 3,3′‐diaminobenzidin (DAB, Vector, Burlingame, USA). The sections were subsequently subjected to haematoxylin for counterstaining. Images were captured in each section at 40× magnifications for up to 15 non‐overlapped fields. The percentage of positively stained areas in each field was analysed using Image J software. The average of the percentage was calculated in all analysed fields of both left and right kidneys of each animal.

### 2.10 Sirius red staining

The deposition of extracellular matrix in the interstitial area of the cortex was evaluated using Sirius red (mainly staining collagen I and III) staining as previously described.^25^ Fiji software was used to quantify the percentage of positively‐stained area of 12–15 captures at 40× per kidney. An average of all percent from both kidneys of each animal was calculated for statistical analysis.

### 2.11 Statistical analyses

Data, presented as bar graphs with the mean ± standard error of the mean (SEM), were analysed using IBM SPSS Statistics v26.0 software. Sample size denotes the number of mice in each study group. Differences among experimental groups were analysed by one‐way ANOVA and LSD test was applied for multiple comparisons. A *p*‐value of less than 0.05 was considered statistically significant.

## 3 RESULTS

### 3.1 Improved kidney function and structure in the 2‐week model

**FIGURE 1:**
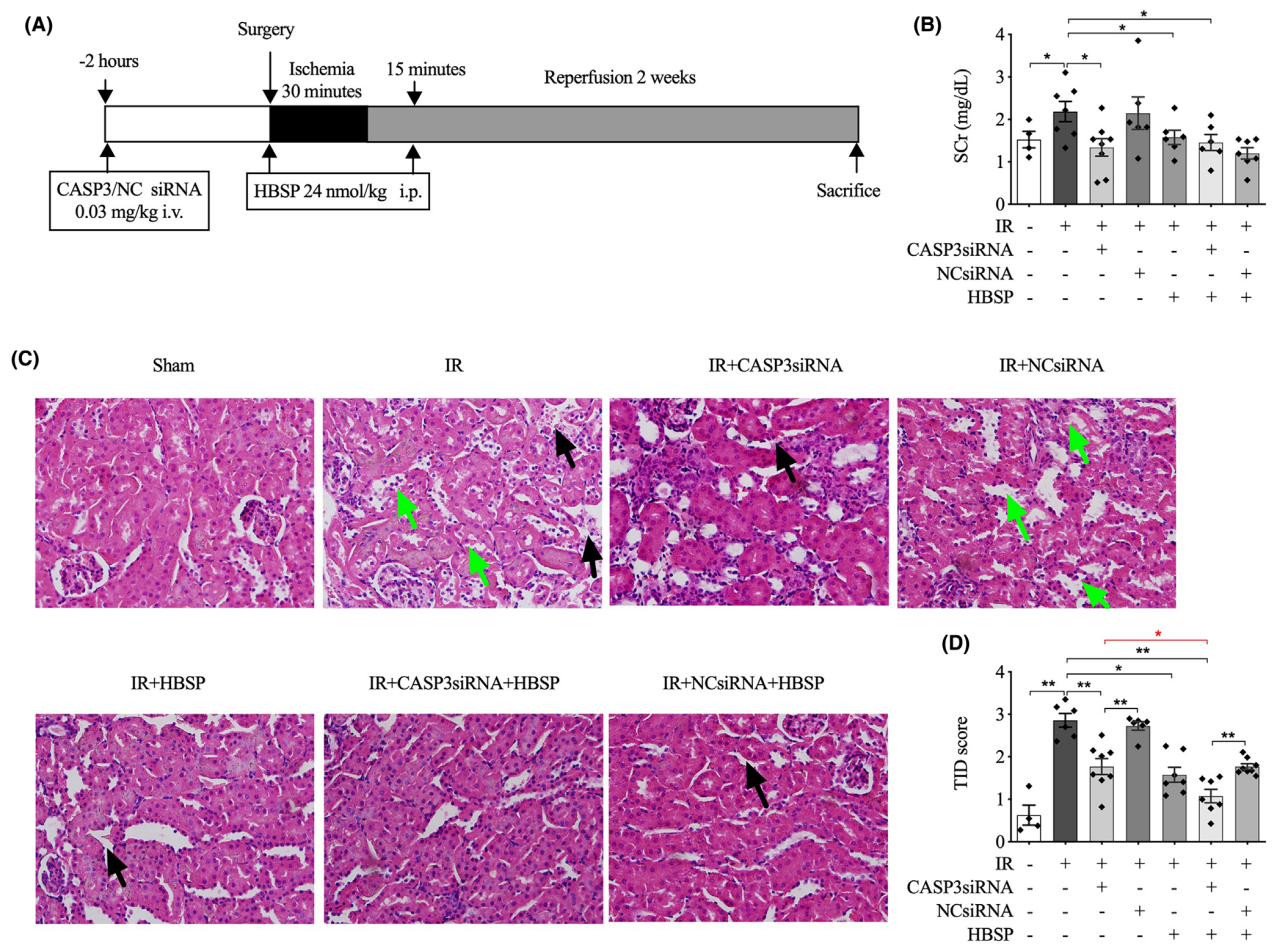
Improved renal function and structure by CASP3siRNA and/or HBSP at 2 weeks post IR. (A) Schematic figure for the animal experimental design; (B) The level of serum creatinine (SCr) was determined by biochemistry test; (C) Representative images of kidney cortex by H&E staining. Damage of tubular epithelium was indicated by green arrows and interstitial dilation by black arrows. (D) Semi‐quantitative scoring of tubulointerstitial damage (TID). Sham: *N* = 4; IR: *N* = 6–7; treatment groups: *N* = 6–8. Data was shown as mean ± SEM, and analysed by one‐way ANOVA and LSD test. *p < 0.05; **p < 0.01.

The design of the 2‐week animal model is illustrated in Figure 1A. The SCr level was significantly raised by IR injury at 2 weeks, but lowered by the administration of CASP3siRNA, HBSP and co‐treatment of CASP3siRNA and HBSP (Figure 1B). In addition, the higher level of SCr was shown in the IR mice treated by NCsiRNA in comparison with CASP3siRNA.

The damage in the cortical areas of kidneys was scored on H&E stained paraffin sections. The dead and sloughing off TECs were shown, as well as the infiltration of inflammatory cells and the expansion of interstitial areas (Figure 1C). The extent of upregulated TID caused by IR injury was significantly decreased by CASP3siRNA, HBSP or their co‐treatment that even further improved TID compared with any single reagent (Figure 1D). Additionally, a significant lower TID score was revealed in IR or HBSP‐modified IR kidneys treated by CASP3siRNA compared with NCsiRNA.

### 3.2 Alleviated apoptosis in the 2‐week model

**FIGURE 2:**
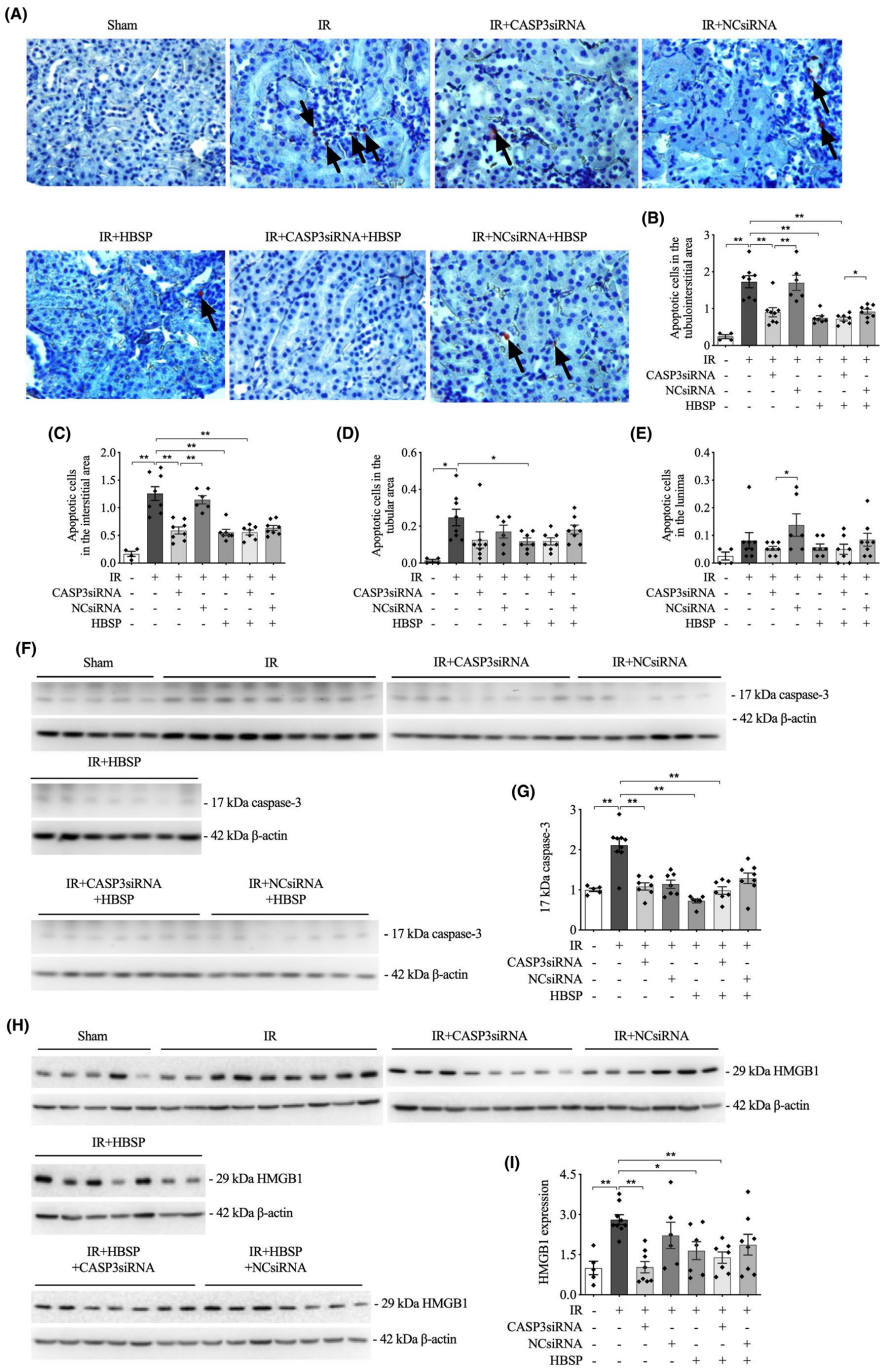
Ameliorated cell apoptosis and reduced expression of active caspase‐3 and HMGB1 by CASP3siRNA and/or HBSP at 2 weeks post IR. (A) Representative images of apoptotic cells in each indicated group, which were labelled with 3‐amino‐9‐ethylcarbazole (AEC, dark red colour) and pointed by arrows; (B) Total apoptotic cells in the tubulointerstitial area; (C–E) Apoptotic cells in the interstitial, tubular and lumina area, respectively. (F) Representative western blots of active 17 kDa caspase‐3 protein; (G) Semi‐quantitative analysis of the expression of 17 kDa caspase‐3 protein, corrected by the endogenous control β‐Actin; (H) Representative western blots of HMGB1 protein; (I) Semi‐quantitative analysis of the expression of HMGB1 protein, corrected by the endogenous control β‐Actin. Sham: *N* = 4/5; IR: *N* = 8/9; treatment groups: *N* = 6–8. Data was shown as mean ± SEM, and analysed by one‐way ANOVA and LSD test. *p < 0.05; **p < 0.01.

To determine the effect of early administration of CASP3siRNA and HBSP on cellular apoptosis at 2 weeks post IR, in situ staining of fragmented DNA in apoptotic cells were performed. Representative images of apoptotic cells were demonstrated in the indicated groups (Figure 2A). The total number of positively stained apoptotic cells in the cortical tubulointerstitial area was greatly raised by IR, but lowered by CASP3siRNA, HBSP and co‐treatment of CASP3siRNA and HBSP (Figure 2B). In contrast to the NCsiRNA control, CASP3siRNA reduced apoptosis in either IR kidneys or HBSP‐modified IR kidneys. The change trend of positive cells in the interstitial area shared a similarity with that in the tubulointerstitial area (Figure 2C). In the tubular area, the number of positive cells was increase by IR injury, but reduced by HBSP significantly (Figure 2D). However, in the lumina, there were no significant changes between the sham and IR groups or IR groups and treatment groups (Figure 2E).

### 3.3 Decreased active caspase‐3 and HMGB1 protein in the 2‐week model

The levels of active caspase‐3 and HMGB1 proteins, markers of tissue injury and inflammation, were investigated in this 2‐week IR model. The expression of active 17 kDa caspase‐3 in the kidneys detected by western blotting was significantly increased by IR injury (Figure 2F,G), but was lowered down by CASP3siRNA, HBSP and co‐treatment. Similarly, HMGB1 expression in the kidneys was upregulated by IR (Figure 2H,I), but downregulated by the treatment of CASP3siRNA, HBSP and co‐treatment.

### 3.4 Increased PCNA but decreased α‐SMA expression in the 2‐week model

**FIGURE 3:**
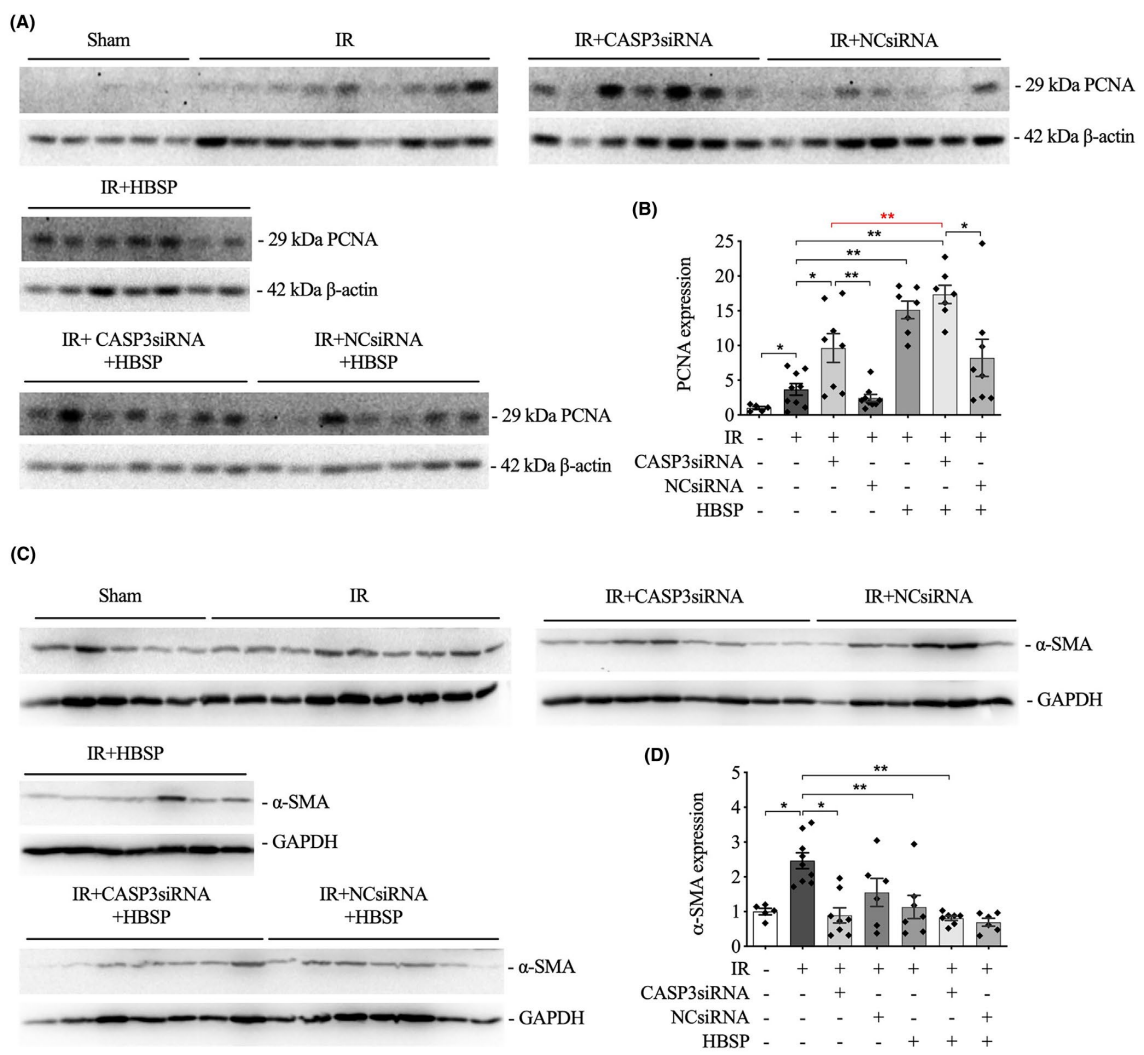
Raised expression of PCNA but decreased expression of α‐SMA by CASP3siRNA and/or HBSP at 2 weeks post IR. (A) Representative western blots of PCNA protein; (B) Semi‐quantitative analysis of the expression of PCNA protein, corrected by the endogenous control β‐Actin. (C) Representative western blots of α‐SMA protein; (D) Semi‐quantitative analysis of the expression of α‐SMA protein, corrected by the endogenous control GAPDH. Sham: *N* = 4/5; IR: *N* = 8/9; treatment groups: *N* = 7–8. Data was shown as mean ± SEM, and analysed by one‐way ANOVA and LSD test. *p < 0.05; **p < 0.01.

In contrast to the sham controls, the expression of PCNA protein was significantly triggered by IR as demonstrated by western blotting detection (Figure 3A,B). The level of PCNA was further increased by the treatment of CASP3siRNA or HBSP. Moreover, co‐treatment of CASP3siRNA and HBSP further increased PCNA expression compared with single CASP3siRNA treatment. In contrast to the corresponding NCsiRNA controls, a significant higher expression of PCNA was induced by CASP3siRNA treatment with or without HBSP in mice subjected to IR injury. Immunoblotting results revealed that the level of α‐SMA was upregulated by IR significantly, but decreased by CASP3siRNA and co‐treatment of CASP3siRNA and HBSP (Figure 3C,D).

### 3.5 Reduced collagen I and decreased Sirius red staining in the 2‐week model

**FIGURE 4:**
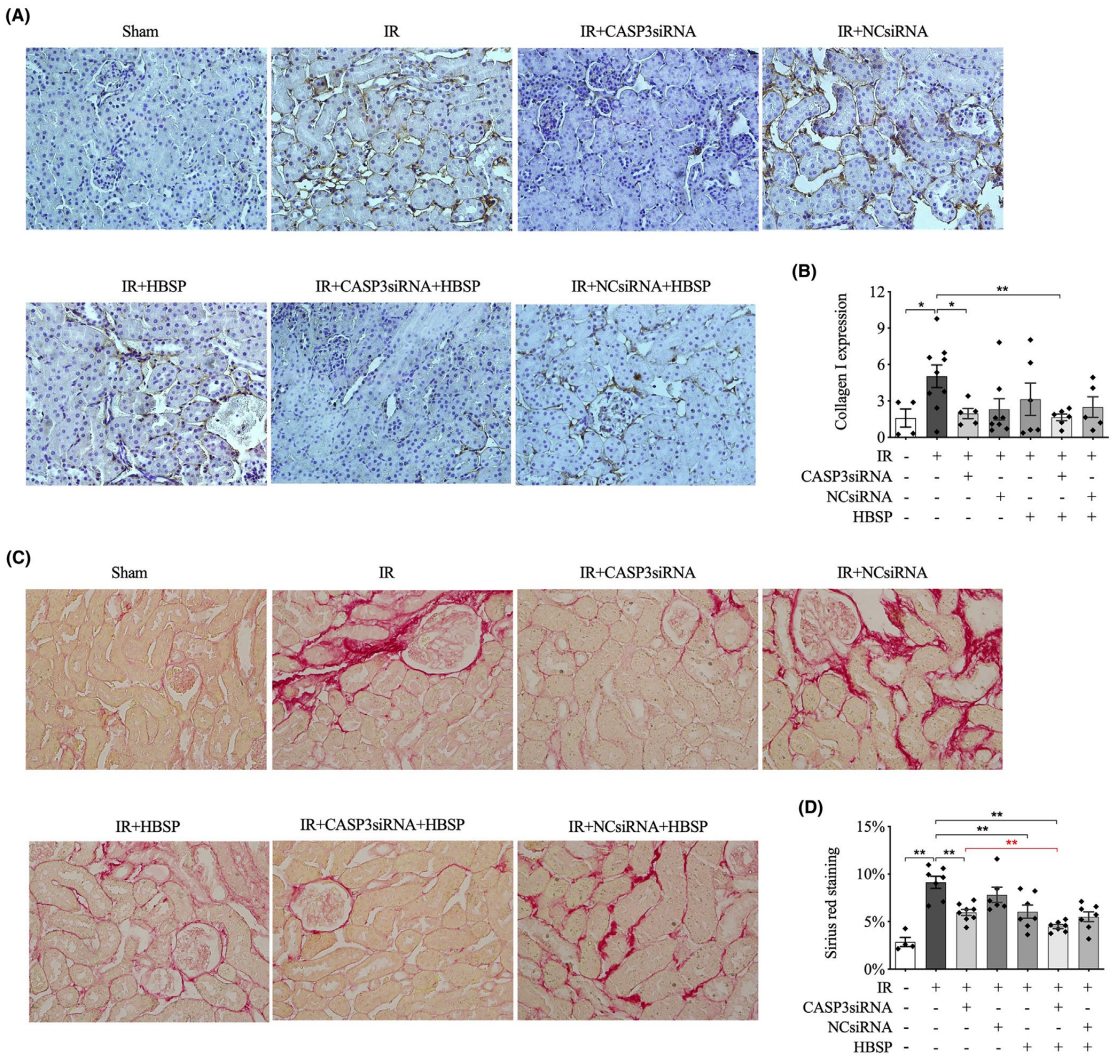
Decreased expression of collagen I and Sirius red staining areas in renal cortex by CASP3siRNA and/or HBSP at 2 weeks post IR. (A) Immunohistochemistry staining of collagen I, visualized by 3,3′‐diaminobenzidin (DAB); (B) Semi‐quantitative analysis of the expression of α‐ collagen I protein. (C) Representative micrographs of Sirius red staining in each group; (D) Semi‐quantitative analysis of the positive staining area of Sirius red. Sham: *N* = 4; IR: *N* = 8; treatment groups: *N* = 6–8. Data was shown as mean ± SEM, and analysed by one‐way ANOVA and LSD test. *p < 0.05; **p < 0.01.

To test the development of kidney fibrosis, the deposition of collagen I and various collagens revealed by Sirius red staining in the interstitial area were evaluated. The positively stained area of collagen I was greatly increased by IR, which was decreased by CASP3siRNA and co‐treatment of CASP3siRNA and HBSP (Figure 4A,B). Compared with sham controls, the area of Sirius red staining was significantly raised by IR which was greatly reduced by HBSP, CASP3siRNA and their co‐treatment (Figure 4C,D). In addition, a lower area of Sirius red staining was exhibited in IR mice with co‐treatment of HBSP and CASP3siRNA in contrast to those treated with CASP3siRNA only.

### 3.6 EPOR and EPOR/ *β*cR in the 2‐week model

**FIGURE 5:**
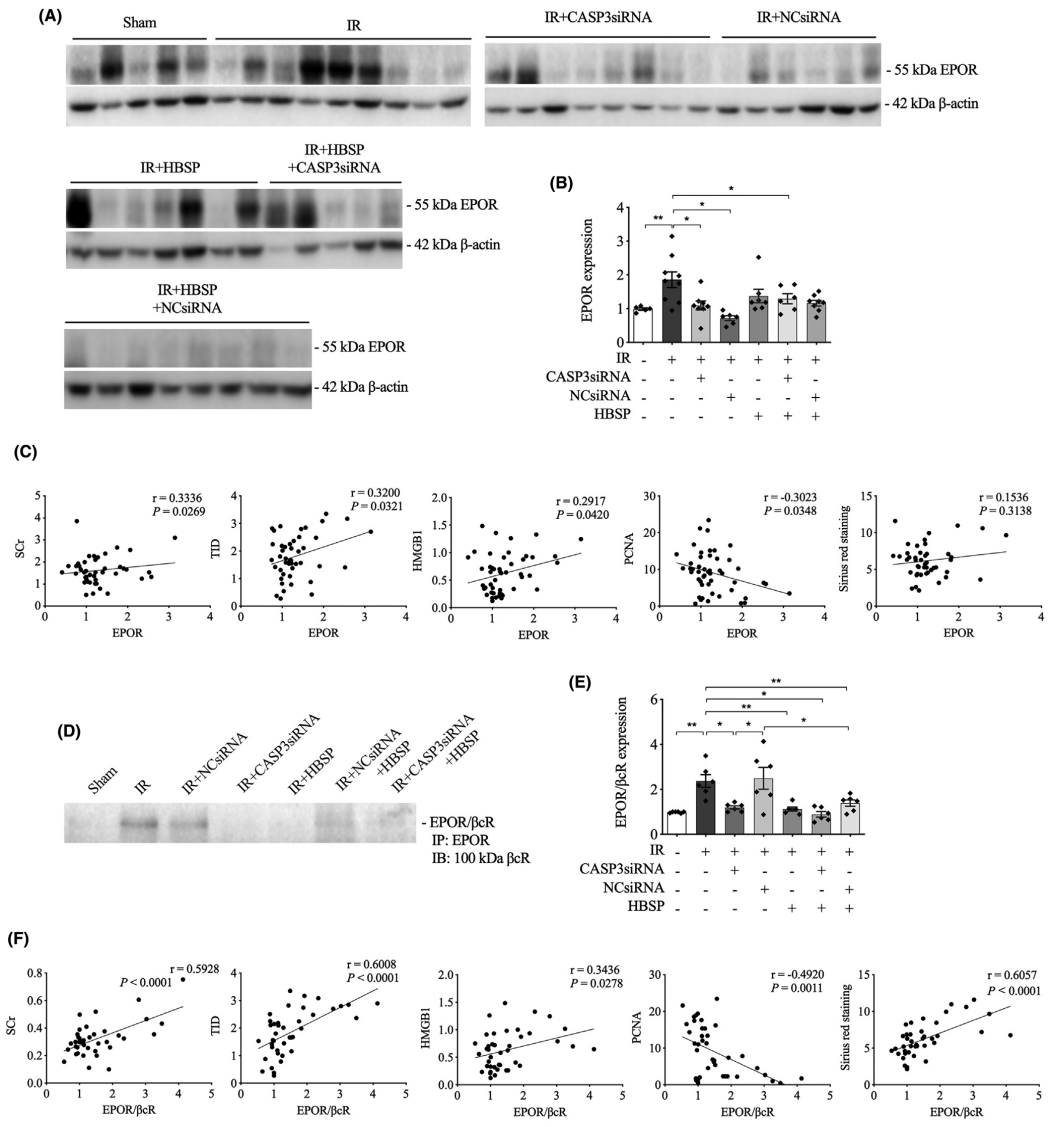
Decreased renal expression of EPOR and EPOR/βcR by HBSP and/or CASP3siRNA at 2 weeks post IR. (A) Representative western blots of EPOR protein; (B) Semi‐quantitative analysis of the expression of EPOR protein, corrected by the endogenous control β‐Actin; (C) The linear correlation between EPOR expression and levels of SCr, TID, HMGB1, PCNA and Sirius red staining. (D) Representative immunoblots of βcR that co‐immunoprecipitated with EPOR representing EPOR/βcR; (E) Semi‐quantitative analysis of the expression of EPOR/βcR heterodimer; (F) The linear correlation between EPOR/βcR expression and levels of SCr, TID, HMGB1, PCNA and Sirius red staining. Data was shown as mean ± SEM, and analysed by one‐way ANOVA and LSD test. *p < 0.05; **p < 0.01.

The protein expression of EPOR was significantly increased by IR, but reduced by CASP3siRNA and co‐treatment of HBSP and CASP3siRNA (Figure 5A,B). However, the level of EPOR in IR kidneys was also decreased by NCsiRNA. Correlation analysis showed that the level of EPOR was positively correlated with SCr, TID, apoptosis, HMGB1 and negatively correlated with PCNA, but not correlated with Sirius red staining (Figure 5C).

The expression of heterdimer receptor EPOR/*β*cR was also elevated by IR at 2 weeks, which was decreased by the treatment of CASP3siRNA, HBSP and their co‐treatment, as well as NCsiRNA + HBSP (Figure 5D,E). The level of EPOR/*β*cR in IR kidneys was significantly decreased by CASP3siRNA treatment in contrast to the NCsiRNA. The expression of EPOR/*β*cR was greatly decreased by HBSP in NCsiRNA‐treated IR kidneys. The level of EPOR/*β*cR was positively correlated with SCr, TID, HMGB1 and Sirius red staining but negatively correlated with PCNA (Figure 5F).

### 3.7 Apoptosis and HMGB1 expression in kidney epithelial cells

**FIGURE 6:**
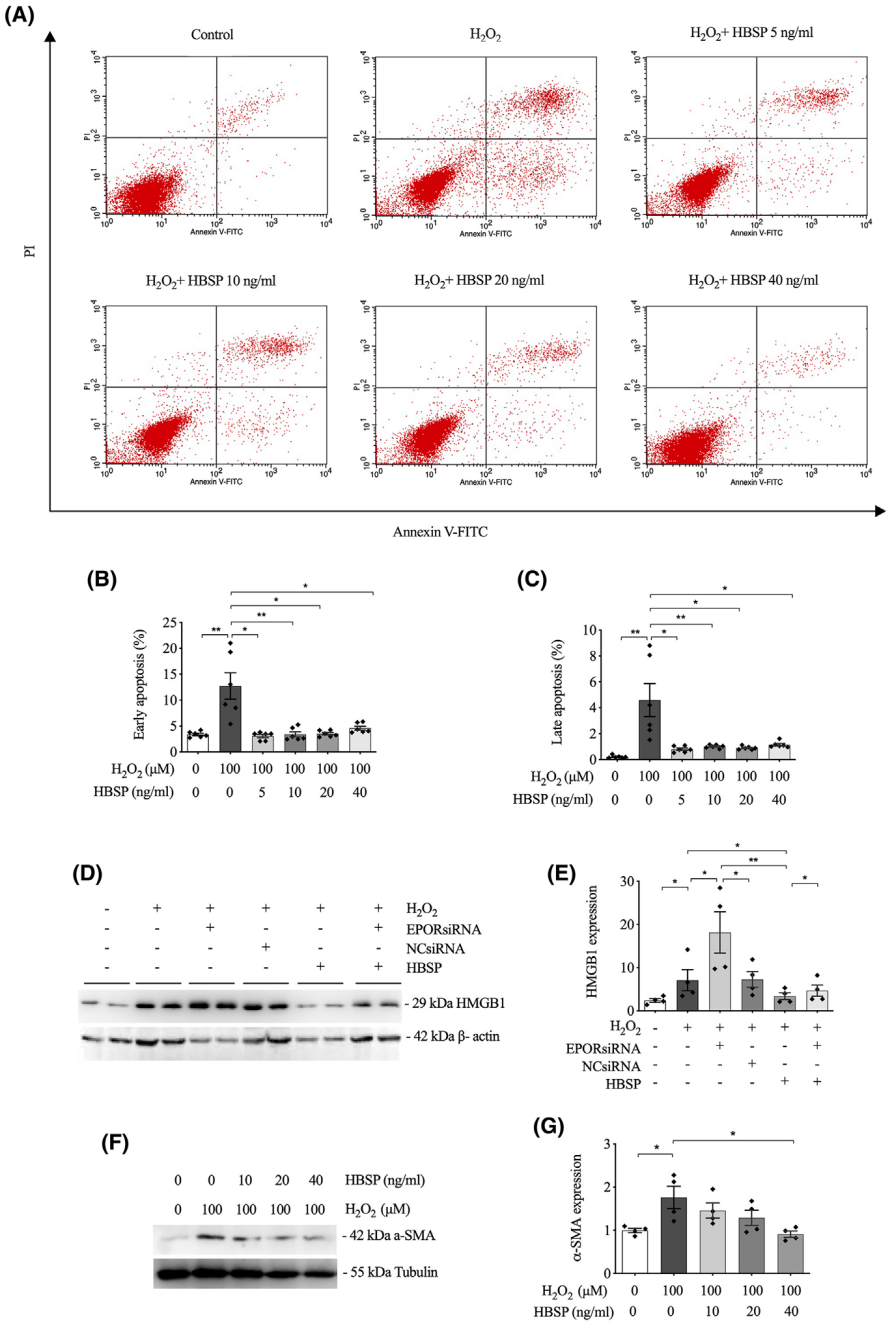
HBSP reduced tubular cell apoptosis, HMGB1 and α‐SMA expression induced by hydrogen peroxide. (A) Representative flow cytometry detection of early apoptosis (lower right quadrant) and late apoptosis (upper right quadrant) of TCMK‐1 cells treated with H_2_O_2_ 100 μM and different concentrations of HBSP from 0 to 40 μg/mL at 24 h; (B, C) Analysis of early and late apoptotic cell rates; (D) Representative western blots of HMGB1 protein; (E) Semi‐quantitative analysis of the expression of HMGB1 protein, corrected by the endogenous control β‐Actin. (F) Representative western blots of α‐SMA protein; (G) Semi‐quantitative analysis of the expression of α‐SMA, corrected by the endogenous control Tubulin. Three independent experiments. Data was shown as mean ± SEM, and analysed by one‐way ANOVA and LSD test. *p < 0.05; **p < 0.01.

Both early and late apoptotic cells of TCMK‐1 triggered by H_2_O_2_ stimulation at 24 h were significantly decreased by HBSP treatment at 5, 10, 20 and 40 ng/mL (Figure 6A–C). The expression of injury marker HMGB1 was increased by H_2_O_2_ but significantly reduced by additional HBSP treatment (Figure 6D,E). The effect of EPORsiRNA was previously verified to downregulate the expression of EPOR protein in TCMK‐1 cells significantly.^24^ Under H_2_O_2_ stimulation, the level of HMGB1 was further enhanced by EPORsiRNA intervention significantly in contrast to the NCsiRNA. In addition, the level of HMGB1 was increased by combined HBSP and EPORsiRNA intervention compared to the HBSP treatment alone.

### 3.8 α‐SMA changed in kidney epithelial cells

The level of α‐SMA protein was significantly increased by H_2_O_2_ in TCMK‐1 cells at 48 h after stimulation (Figure 6F,G). However, the level of α‐SMA was gradually decreased by the treatment of HBSP at 10, 20, 40 ng/mL, which reached statistical significance at 40 ng/mL.

## 4 DISCUSSION

**FIGURE 7:**
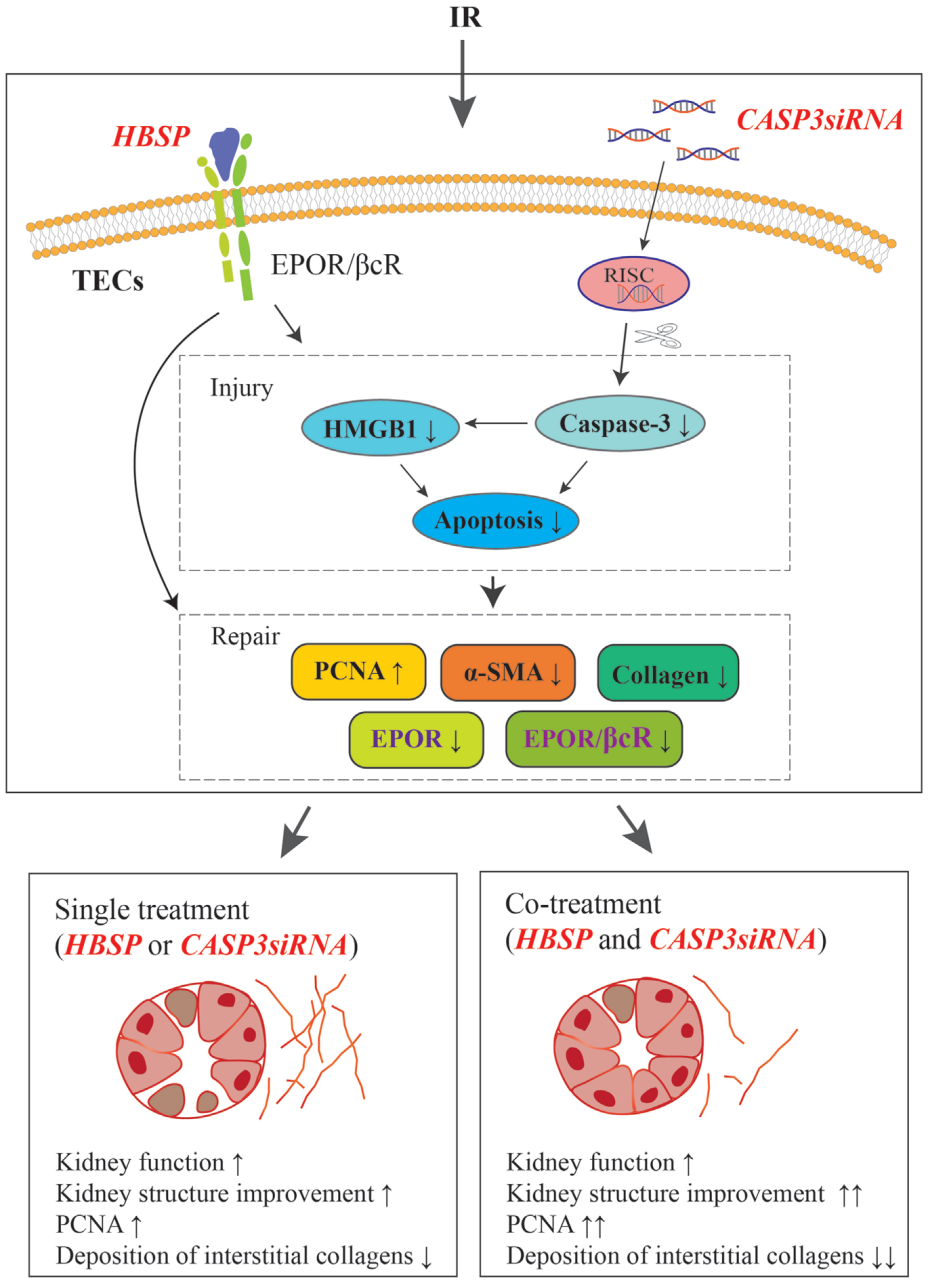
Schematic illustration of the advantage of co‐treatment with HBSP and CASP3siRNA. HBSP specifically recognizes EPOR/βcR on cellular membranes, while CASP3siRNA forms RNA‐induced silencing complex (RISC) in the cytoplasm that specifically downregulates caspase‐3 mRNA. In the present study, single treatment with HBSP or CASP3siRNA significantly preserved kidney function and structure by ameliorating kidney injury markers including active caspase‐3, HMGB1 and cellular apoptosis, and promoting repair by upregulating PCNA and downregulating fibrotic‐related markers including α‐SMA and collagens. The upregulated expression of EPOR and EPOR/βcR by IR injury was subsequently reduced by any single treatment. However, EPOR/βcR is a better indicator than (EPOR)_2_ in monitoring the responses of the treatment. Most importantly, co‐treatment with HBSP and CASP3siRNA further improved kidney structure, PCNA expression and interstitial collagen deposition compared to a single treatment of CASP3siRNA.

The treatment of AKI is still challenging in not only its recovery, but also its transition to CKD and continuous progression to fibrosis. In this study, early treatment by either CASP3siRNA or EPO‐derived HBSP effectively protected the IR kidneys at 2 weeks, in terms of decreasing SCr, structural damage and injury biomarkers, along with increasing cell proliferation, but reducing myofibroblast markers and collagen deposition. Co‐treatment of CASP3siRNA and HBSP showed the even better preservation of tubulointerstial structure, further enhanced cell proliferation markers and reduced the deposition of collagens in the interstitial area compared to single treatment of CASP3siRNA. These results suggest that early co‐treatment with CASP3siRNA and HBSP has synergistic benefits on reducing injury, promoting repair and preventing the progression of AKI‐to‐CKD post IR as summarised in a schematic illustration (Figure 7).

IR resulted in continuous kidney injury over 2 weeks, with elevated serum creatinine and TID along with highly expressed injury and fibrotic markers. Here, silencing caspase‐3 significantly preserved kidney function and structure at 2 weeks after IR, which may explain the regulated cellular apoptosis, the secondary necrosis revealed by the reduced HMGB1 and fibrosis, but increased PCNA expression. Previous reports show that knockout of caspase‐3 in mice attenuated kidney microvascular injury by reducing microvascular congestion and preserving peritubular endothelial cell markers and their fenestration from Day 3 after IR injury and lasted for 3 weeks, with decreased α‐SMA expression, collagen deposition and kidney fibrosis.^26^ This suggests that silencing caspase‐3 makes priority on ameliorating injury and promoting repair after IR‐induced AKI.

Caspase‐3 is also an important molecule regulating tissue regeneration. The present study shows that silencing caspase‐3 increased the expression of PCNA in IR kidneys at 2 weeks, which has also been demonstrated in B cells with caspase‐3‐deficiency that promotes their proliferation by stimulating PCNA and cell cycles.^27^ It might result from the lowered level of damage by reducing the kidney apoptosis, while it might benefit from the survived cells facilitating re‐constructing kidney structure.^28^ In addition, it was demonstrated that the role of CASP3siRNA on elevating the kidney expression of secretory leukocyte peptidase inhibitor (SLPI) and serpin family A member 3 (SERPINA3) shown in the global genomic profiles at 48 h after renal IR injury.^23^ The expression of SLPI and SERPINA3 were also increased in human transplanted kidneys as early as 30 min after surgery, indicating their role of early diagnosis and involvement in the IR‐related injury.^29^ The induction of SLPI‐expressing vectors in kidney tubular proximal epithelial cells greatly increased the abundance of cyclin D1 and the number of proliferating cells.^30^ In addition, SERPINA3 was essential for the proliferation of neural stem cells^31^ and aortic smooth muscle cells,^32^ indicating its role on repair. Moreover, SERPINA3 expression detects kidney fibrosis and it could be a biomarker for the early detection of AKI to CKD.^33^ The above genes regulated at the early stage of IR‐induced AKI could provide potential mechanisms for highly‐expressed kidney PCNA at the repair stage of 2 weeks. Administration of HBSP had a stronger effect on promoting PCNA expression than that of CASP3siRNA and combined treatment of the two further increased PCNA levels and reduced TID score for 2 weeks post IR. HBSP also showed its role on regulating proliferation through multiple pathways including involving Src and PI3K/Akt signalling in endothelial cells,^34^, ^35^ and Sonic hedgehog (Shh)/Gli Family Zinc Finger 1 (Gli1) signalling pathways on neural progenitor cells,^36^ while the latter pathway was also mediated in kidney regeneration after kidney IR injury.^37^ The proliferative‐associated mechanism and signalling pathways involved in IR kidneys treated with CASP3siRNA and/or HBSP remains elucidated and further investigation, in particular the mechanism of CASP3siRNA and HBSP or HBSP alone may be involved in limiting injury, promoting repair, and preventing the transition of AKI to CKD.

Incomplete repair, characterized as progressing fibrosis, is a major feature during AKI progression to CKD, contributing to the poor prognosis of the IR injured kidney. Previously, we reported the synergistic effect of CASP3siRNA and CHBP (cyclic HBSP with better stability) on further preservation of kidney structure after IR 48 h by downregulating angiopoietin like 2 (ANGPTL2) and upregulating gremlin 1 (GREM1).^23^ ANGPTL2 was essential for the progression of kidney fibrosis in mouse unilateral ureteral obstruction‐induced AKI by increasing TGF‐β expression.^38^ GREM1 activates endothelial cells to induce angiogenesis that is essential for the maintenance of the functions of microvasculature, thus ameliorating tubular damage and promoting repair.^39^, ^40^ The above evidences supported our findings on that CASP3siRNA and/or HBSP significantly decreased the level of α‐SMA expression and interstitial deposition of collagen in the present kidney IR models at 2 weeks, which was also coordinated with our previous findings.^41^ In vitro, HBSP also showed a significant reduction of α‐SMA in TECs induced by H_2_O_2_. The decreased expression of α‐SMA could represent limited myofibroblast proliferation and tubular epithelial‐mesenchymal‐transition. Collagen I, mainly secreted by fibroblasts, is the primary collagen type of the tubulointerstitial extracellular matrix and reduced production by CASP3siRNA and its combined treatment with HBSP but not HBSP only. During renal fibrosis, the production and deposition of other collagens mainly including collagen III and IV can also be increased in the tubular basement membrane and interstitial areas.^42^ All these collagens stained by Sirius red was revealed significant reduction by CASP3siRNA and furthered by additional HBSP treatment. It is suggesting CASP3siRNA would modify various collagen production but HBSP mainly modifies collagens such as type III and IV, as showed its targeting cells are mainly TECs that contribute to the production of collagens IV.^24^ The effects of CASP3siRNA and HBSP on reducing kidney fibrosis after IR could provide new concept on therapeutic strategy for IR‐induced AKI‐CKD transition.

Based on CASP3siRNA treatment, additional administration of HBSP further reduced TID score, increased PCNA expression and reduced collagen deposition at 2 weeks after IR. This may be attributed to the reduced level of injury showed by decreased apoptotic TECs and HMGB1 expression compared to HBSP treatment alone. This synergistic co‐effect may be also associated with the expression of receptor EPOR, which was regulated by IR and inflammatory factors.^43^ In vitro cultured kidney epithelial cells, the raised EPOR by H_2_O_2_ and its knockdown by siRNA were closely related to cellular apoptotic death^24^ and HMGB1 expression. In this in vivo model, it has been also shown that the expression of EPOR was correlated with kidney injury parameters such as SCr and HMGB1, so as the EPOR/βcR. Therefore, EPOR/βcR is not only associated with the initiation of repair, but also reflects the degree of kidney injury. Its expression was swiftly upregulated upon IR,^44^ indicating a critical treatment window for HBSP at the onset of IR injury and the administration of HBSP at a later time point of IR will be much less effective. EPOR also forms homodimers, (EPOR)_2_, that is erythropoietic and could contribute to the progression of kidney fibrosis after IR.^16^, ^45^ Only EPOR/βcR, but not EPOR, was positively correlated with Sirius red staining that stains various collagens, indicating EPOR/βcR is more crucial than (EPOR)_2_ in monitoring the responses of the treatment. HBSP treatment significantly reduced the expression of EPOR/βcR, but not that of EPOR. Nevertheless, the high level of EPOR in IR kidneys was numerically reduced by CASP3siRNA and its co‐treatment with HBSP. Thus, the co‐administration of CASP3siRNA and HBSP may contribute to the reduction of EPOR, and the prevention of kidney fibrosis and the recovery of IR injury. Our previous study on the combined therapy of CASP3siRNA and CHBP (the cyclic form of HBSP with longer half‐life in the serum^22^) was observed at 48 h after kidney IR injury that showed the co‐treatment significantly reduced the expression of active 17 kDa caspase‐3 and HMGB1 as well as preserved kidney structure in comparison with single CASP3siRNA treatment or NCsiRNA and CHBP treatment.^23^ However, some significantly changed parameters including active caspase‐3 and HMGB1 expression, as well as other differentially expressed genes, at 48 h might be not significant at 2 weeks, but its impact could be continued in long term reflected by changes in renal function and structure. It is worth noting that early modification by combined HBSP and CASP3siRNA could provide sustained protective effects on IR‐injured kidneys to the latter stage.

There are some limitations to this study. An animal model with a longer reperfusion time such as 8 and 12 weeks could be performed to further confirm the effect of combined treatment of CASP3siRNA and HBSP on preventing chronic kidney injury after renal IR injury‐induced AKI. As the diverse aetiology of clinical AKI, the effect of this co‐treatment could be further explored in other AKI models such as unilateral ureteral obstruction or lipopolysaccharide‐induced AKI models to verify its advantages compared to either single treatment. The long‐term renoprotective effect of CASP3siRNA and HBSP could be further differentiated to explore the mechanism of synergistic effect of combined treatments on the progression of kidney fibrosis.

## 5 CONCLUSIONS

Combined treatment of CASP3siRNA and peptide HBSP has greater protective effects than single agent on limiting injury and promoting repair after renal IR injury, especially on improving tubulointerstial structure, cell proliferation and collagen deposition. This finding provides a new evidence for potential therapeutic strategy in preventing the chronic progression and fibrosis of IR‐induced AKI.

## AUTHOR CONTRIBUTIONS

**Yuanyuan Wu:** Data curation (equal); formal analysis (equal); funding acquisition (lead); writing – original draft (lead). **Yanan Wang:** Data curation (equal); formal analysis (equal); methodology (equal); validation (equal). **Fei Chen:** Data curation (equal); formal analysis (equal); methodology (equal); software (equal). **Cheng Han:** Software (equal); validation (equal). **Lili Huang:** Project administration (equal); validation (equal). **Wenli Sai:** Resources (equal). **Yaping Fan:** Supervision (supporting). **Nigel J. Brunskill:** Supervision (supporting); writing – review and editing (supporting). **Bin Yang:** Conceptualization (lead); funding acquisition (lead); project administration (lead); supervision (lead); writing – review and editing (lead).

## CONFLICT OF INTEREST STATEMENT

The authors declare no conflict of interest.

## Data Availability

The data that support the findings of this study are available from the corresponding author upon reasonable request.
